# Sleep phenotypes and clinical outcomes in individuals with chronic obstructive pulmonary disease: A cohort study

**DOI:** 10.1007/s11325-026-03683-2

**Published:** 2026-04-25

**Authors:** Daniele Dala Pola, Thaiuana Maia Ferreira, Elis Moraes Martins, Leticia Yumi Ogochi, Ana Lívia Trindade, Giovanna Freitas, Maria Gabriela Fernandes, Arthur Eumann Mesas, Raquel Hirata, Fabio Pitta

**Affiliations:** 1 Laboratory of Research in Respiratory Physiotherapy, Health Sciences Center, Universidade Estadual de Londrina, Londrina, Brazil; 2 Health and Social Research Center, Universidad de Castilla-La Mancha, Cuenca, Spain

**Keywords:** Chronic obstructive pulmonary disease, Sleep, Muscle strength, Physical activity in daily life, Exercise capacity, Mental disorders

## Abstract

**Introduction:**

In COPD, most studies have examined sleep characteristics in isolation, and limited evidence exists regarding sleep phenotypes and their impact on clinical outcomes.

**Objective:**

To prospectively investigate the impact of sleep phenotypes on several relevant clinical outcomes in individuals with COPD.

**Methods:**

Based on sleep nighttime assessed at baseline by both objective and subjective methods, individuals were classified into different sleep phenotypes: ‘short sleep’ and ‘high propensity to sleep + average sleep’. Furthermore, in a prospective cohort, individuals were assessed at baseline and after 12 months for lung function, body composition, peripheral muscle strength, functional capacity, daily physical activity (PA) and symptoms of dyspnea, anxiety, and depression.

**Results:**

Twenty-nine individuals with COPD were analyzed. In both subjective and objective assessments, the ‘short sleep’ phenotype showed worsening in more outcomes when compared to the other phenotype, with significant worsening in FEV_1_ (*p* = 0.038), dyspnea (*p* = 0.020), light-intensity PA as % of the day (*p* = 0.028), sedentary time as % of the day (*p* = 0.028) and peripheral muscle strength (*p* = 0.003). Repeated-measures ANOVA showed a significant main effect of time for light PA and sedentary time, with large effect sizes. There was no significant interaction between time and subjective sleep phenotypes, although sedentary time showed a borderline result.

**Conclusion:**

Individuals with COPD who present the ‘short sleep’ phenotype show significant worsening in lung function, daily PA and peripheral muscle strength over 12 months. Furthermore, the interaction between sleep duration and phenotype may influence physical activity and sedentary behavior.

**Supplementary Information:**

The online version contains supplementary material available at 10.1007/s11325-026-03683-2.

## Introduction

It is well known that sleep disorders are prevalent dysfunctions among individuals with chronic obstructive pulmonary disease (COPD), ranking just behind dyspnea and fatigue [1]. This reality is comprehensible, as these individuals face a respiratory biomechanical disadvantage that becomes more pronounced during sleep [2].

Sleep deprivation in patients with COPD is associated with poorer clinical and functional outcomes [3–5], as well as an increased risk of exacerbations and mortality [6]. Despite this scientific background, most studies have focused primarily on sleep quality and quantity. However, other sleep parameters are also relevant, and a more comprehensive approach that considers multiple outcomes simultaneously may better characterize individual sleep patterns. One strategy that enables this multidimensional analysis is the identification of sleep phenotypes.

Sleep phenotypes refer to observable and measurable characteristics that may include sleep duration, quality, regularity, chronotype, and other related aspects. Analyzing sleep phenotypes offers important advantages, such as enabling targeted interventions and the development of novel treatments that promote health from a broader perspective [7].

In sleep-related conditions such as insomnia and obstructive sleep apnea (OSA), the literature on sleep phenotypes has progressed more rapidly [8–9]. In the elderly population, three phenotypes have been identified, namely, ‘average sleep’, ‘insomnia with short sleep’ and ‘heightened sleep propensity’, which were linked to higher mortality [7]. But the investigation of sleep phenotypes in individuals with COPD has been minimally explored to date.

To the best of the authors’ knowledge, only one study has examined sleep phenotypes in COPD and their relationship with mortality [10]. Despite the study’s relevance and innovative approach, the sleep variables used have cutoff points that differ from those recommended by the National Sleep Foundation. Furthermore, age and presence of comorbidities were the most significant factors in differentiating phenotypes, which restricts their application in clinical sleep practice. Moreover, the scientific literature on this important population still lacks studies that combine subjective and objective sleep phenotyping, as well as studies that include more detailed and clinically relevant functional outcomes and that adopt a prospective 12-month design. Therefore, the aim of this study was to prospectively investigate the influence of sleep phenotypes on relevant outcomes such as lung function, body composition, peripheral muscle strength, functional exercise capacity, daily physical activity (PA) and symptoms of dyspnea, anxiety and depression in individuals with COPD.

## Methods

This prospective cohort study was conducted from September 2022 to September 2024 with a convenience sample of individuals with COPD recruited from the community and the Physiotherapy outpatient clinic at the Hospital of State University of Londrina (UEL, Brazil). This study was approved by the institutional Research Ethics Committee (Protocol n.3.471.646) and written informed consent was obtained from all participants.

An initial assessment (A1) was followed by quarterly telephone calls and a final assessment (A2) performed 12 months after A1. All assessments were conducted at UEL’s Laboratory of Research in Respiratory Physiotherapy, and the same researchers performed the evaluations at the two time points.

Inclusion criteria were: COPD diagnosed according to the GOLD [11]; no infections or exacerbations in the last three months; and no severe comorbidities that could potentially interfere with the assessments (e.g., unstable heart disease, cancer treatment, inability to walk independently or severe cognitive impairment). Exclusion criteria were: exacerbation during the period of face-to-face assessments; presence of unstable comorbidities in the final assessment; loss of contact during the follow-up period; or death. The use of hypnotic and/or sedative medications, nocturnal oxygen therapy or positive airway pressure during sleep were not exclusion criteria. The study was conducted and reported following the recommendations of the Strengthening the Reporting of Observational Studies in Epidemiology (STROBE).

### Assessments

Clinical data were collected and other assessments included pulmonary function (spirometry), body composition (bioelectrical impedance analysis), daily PA (ActiGraph wGT3X-BT accelerometer), peripheral muscle strength (dynamometry) and functional exercise capacity (4-meter gait speed test at usual speed [4MGS], timed up-and-go test at usual speed [TUG] and sit-to-stand test in the 1-minute protocol [STS]). Screening for anxiety and depression was performed using the Hospital Anxiety and Depression Scale (HADS) and dyspnea was assessed with the Modified Medical Research Council (mMRC) scale. Details of these methods are provided in the supplementary material, while sleep-related assessments are described in detail below.

#### Subjective assessment of sleep

Subjective assessment was performed by the application of the Pittsburgh Sleep Quality Index (PSQI), which assesses sleep quality over the past month. The questionnaire consists of 19 self-rated questions and 5 questions that should be answered by bedmates or roommates. The 19 questions are categorized into 7 components. The sum of all component scores provides an overall score, which varies between 0 and 21, with higher scores indicating worse sleep quality. In particular, component 1 was used as a metric for sleep satisfaction. The item is rated as 0 (very good), 1 (fairly good), 2 (fairly bad), or 3 (very bad), and low satisfaction was defined as a rating ≥ 2 [12].

#### Objective assessment of sleep

Objective assessment of sleep was performed by actigraphy using the Actiwatch (Philips Respironics, Murrysville, USA). The equipment was used on the non-dominant wrist for 24 h on seven consecutive days. During the days while wearing the device, individuals were instructed to fill out a usage diary. An assessment was deemed valid when there were at least 4 available assessment nights.

#### Daytime sleepiness

The Epworth Sleepiness Scale (ESE) was utilized to assess excessive daytime sleepiness across 8 daily scenarios, where the individual rates the likelihood of dozing off in relation to the past month. The total score varies from 0 to 24, with higher scores indicating more sleepiness, and a score exceeding 10 indicating excessive daytime sleepiness [13].

### Sleep phenotypes

**Fig. 1:**
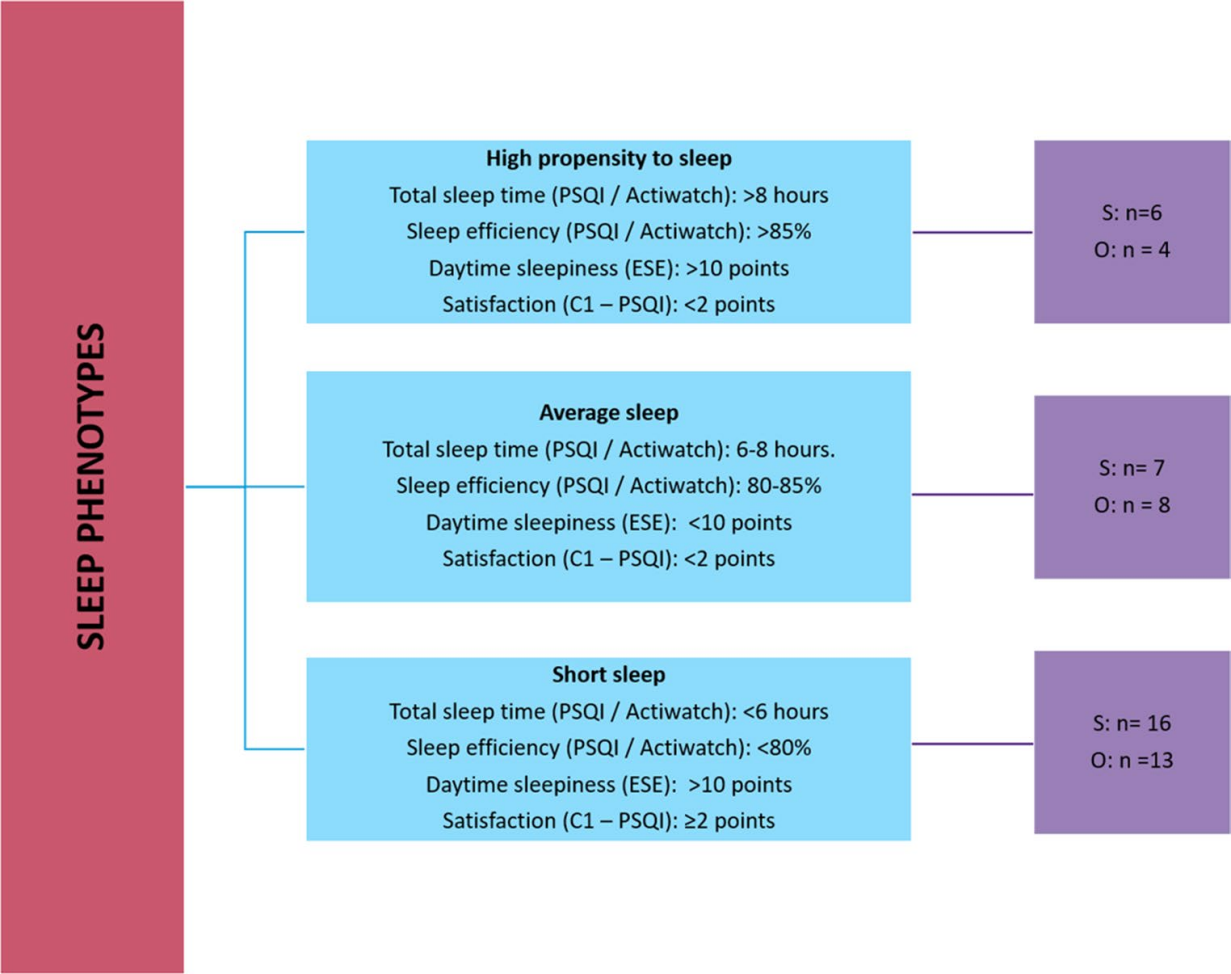
Characterization of sleep phenotypes [7]. The blue column shows a description of each sleep phenotype; the purple column shows the number of individuals in each phenotype, with “S” representing subjective sleep assessment and “O” representing objective sleep assessment. PSQI: Pittsburgh Sleep Quality Index; ESS: Epworth Sleepiness Scale; C1: component 1

As proposed by Wallace et al. [7], individuals were classified into three phenotypes: ‘short sleep’, ‘average sleep’ and ‘high propensity to sleep’. Details and cutoff points used for phenotyping can be found in detail in Fig. 1. In brief, each phenotype was based on baseline assessment of the following variables: total sleep time, sleep efficiency, daytime sleepiness and sleep satisfaction. Each variable has a predefined cutoff point as previously described in the article by Wallace et al. [7] which takes into account the current recommendations from the National Sleep Foundation’s or those proposed by their developers [14]. Initially, phenotypic classification was performed using only subjective sleep data, and objective data (total sleep time and sleep efficiency) were subsequently incorporated. To be classified into a given phenotype, participants were required to meet at least 75% of the predefined criteria, corresponding to at least three of the four evaluated variables. No participants were excluded for failing to meet the proposed classification criteria.

### Statistical analysis

For statistical analysis, normality of data distribution was assessed using the Shapiro-Wilk test. Data distribution were described as mean ± standard or median [interquartile range 25–75%]. For the purpose of statistical comparison, the ‘high propensity to sleep’ and ‘average sleep’ phenotypes were combined into a single phenotype (‘high propensity to sleep + average sleep’) due to the reduced number of subjects in these two subgroups. Comparisons of baseline categorical or continuous data between phenotypes were conducted using the chi-square test or Mann-Whitney U test, respectively. Comparisons within each phenotype (i.e., baseline versus 12 months after) were done using either the dependent samples T-test or Wilcoxon test. Additionally, the change in variables over time, referred to as delta, was calculated (visit 2-visit 1). Deltas were compared between the two phenotypes using the independent samples T-test or Mann-Whitney test. The mixed repeated measures ANOVA was performed to assess the effect of time, where the between-subjects factor represented the sleep phenotypes, and the within-subjects factor was time (two assessment points: baseline and 12 months). Additionally, partial eta squared was calculated in the mixed repeated-measures ANOVA. Finally, for the second assessment time point, the confidence intervals between subjective and objective phenotypes were calculated using the Mann–Whitney test, and the between-group differences were estimated using the Hodges–Lehmann estimator. The SPSS software (version 25.0) and G-Power software (version 3.1) were used, and significance was set as *p* < 0.05.

## Results

**Fig. 2:**
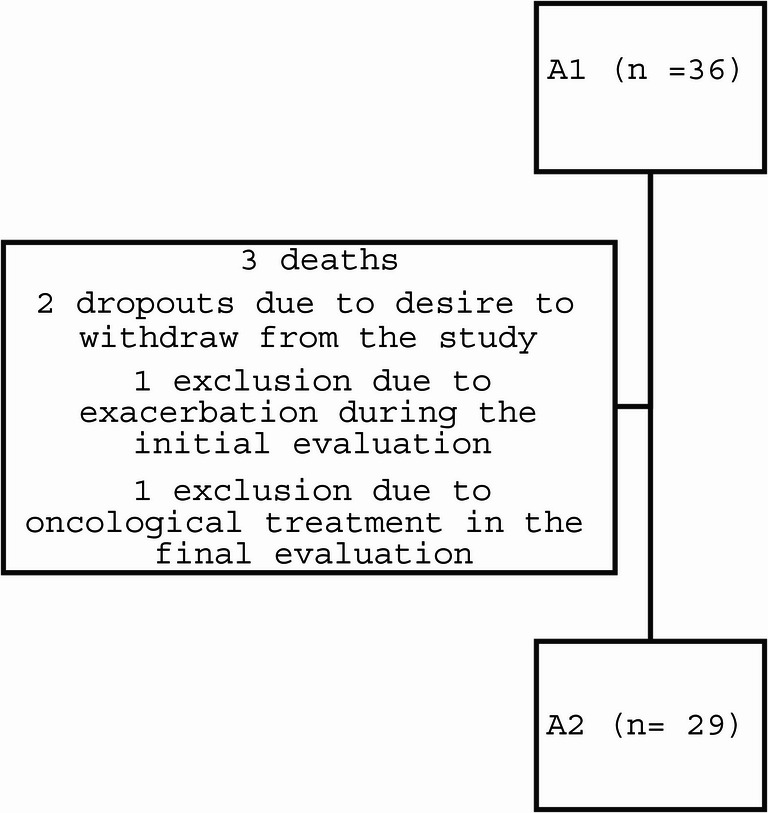
Study flowchart. A1: baseline assessment; A2: assessment after 12 months

Thirty-six individuals were evaluated at baseline, and at the end of the follow-up, 29 were included in the analyses (no imputation was performed). Figure 2 presents the study flowchart. The clinical and demographic characteristics of the sample at A1 are described in Table 1. In general, there was slight predominance of women, with a BMI ranging from normal weight to overweight, mostly white race, married, ex-smokers, with incomplete elementary education and the most frequent comorbidity was systemic arterial hypertension. At the initial assessment, no patients were receiving positive airway pressure therapy or oxygen therapy during the night. By the second assessment, one patient had become dependent on oxygen both during the day and at night.

**Table 1:** Baseline demographic and clinical characteristics of the participants (*n* = 29)

Variable	*n* = 29
Sex, female, *n* (%)	18 (62)
Age, years	68 ± 7
Body mass index, kg/m^2^	27 ± 5
Neck circumference total sample, cm	38 ± 4
Neck circumference men, cm	41 ± 5
Neck circumference women, cm	36 ± 3
Race	
White, *n* (%)	21 (72)
Brown, *n* (%)	6 (21)
Black, *n* (%)	2 (7)
Marital status	
Stable union, *n* (%)	19 (66)
Single, divorced, widower, *n* (%)	10 (34)
Smoking	
Never, *n* (%)	4 (14)
Ex-smoker, *n* (%)	21 (72)
Current smoker, *n* (%)	4 (14)
Pack years (ex-smokers and current smokers)	60 ± 39
GOLD I/II/III/IV, n	3/18/7/1
Education	
Illiterate, n (%)	2 (7)
Incomplete elementary school, *n* (%)	13 (45)
Incomplete high school, *n* (%)	5 (17)
Incomplete higher education, *n* (%)	6 (21)
Complete higher education, *n* (%)	3 (10)
Comorbidities	
Number of exacerbations in the last year, *n*	1 ± 1
Systemic arterial hypertension, yes, *n* (%)	17 (58)
Diabetes mellitus, yes, *n* (%)	6 (21)
Cardiovascular diseases, yes, *n* (%)	6 (21)
Use of inhaled medications	
Long-acting antimuscarinic, yes, *n* (%)	1 (3)
Long-acting beta-agonist, yes, *n* (%)	7 (24)
Inhaled corticosteroid, yes, *n* (%)	4 (14)
Short-acting beta-agonist, yes, *n* (%)	1 (3)

GOLD: Global Initiative for Chronic Obstructive Lung Disease

The comparison of clinical characteristics and potential confounding factors between subjective and objective sleep phenotypes is presented in Table 2, and there were no statistically significant differences.

**Table 2:** Comparison of clinical and sleep variables between subjective and objective phenotypes

Variable	Subjective phenotypes			Objective phenotypes		
	High + average sleep	Short sleep	*p*	High + average sleep	Short sleep	*p*
Sex, man/woman, n	5/8	6/10	0.958	4/8	5/8	0.790
Age, years	70 [67–76]	67 [60–72]	0.329	70 [61–73]	67 [61–72]	0.503
Body mass index, kg/m^2^	27 [24–30]	27 [25–31]	0,746	27 [23–33]	27 [25–29]	0.894
Fat mass index	32 [20–35	29 [21–38]	0.820	31 [21–36]	28 [21–36]	0.684
Active smokers, no/yes, n	13/0	12/4	0.052	11/1	10/3	0.315
Hypnotic medication, no/yes, n	8/5	11/5	0.684	9/3	8/5	0.471
SAH, no/yes, n	5/8	7/9	0.774	5/7	4/9	0.571
DM, no/yes, n	10/3	13/3	0.775	10/2	10/3	0.689
Cardiovascular disease, no/yes, n	7/2	11/4	0.808	8/3	8/3	1.000
Total subjective sleep time, hours	8 [7–10]	5 [4–6]	**< 0.001**	6 [6–9]	6 [5–8]	0.295
Total objective sleep time, hours	7 [6–8]	6 [5–7]	**0.041**	7 [7–8]	5 [5–6]	**< 0.001**
Subjective sleep efficiency, %	83 [72–90]	60 [50–75]	**< 0.001**	78 [66–87]	67 [53–82]	0.152
Objective sleep efficiency, %	80 [70–96]	79 [72–83]	0.373	85 [81–96]	73 [63–78]	**< 0.001**
WASO objective, min	64 [10–104]	82 [52–117]	0.467	59 [10–84]	90 [66–119]	**0.035**
Epworth Sleepiness Scale, points	6 [1–11]	7 [2–10]	0.779	5 [0–10]	7 [3–12]	0.270
PSQI Component 1, points	1 [1–1]	2 [1–2]	**0.004**	1 [1–2]	2 [1–2]	0.320
PSQI score, points	7 [6–8]	12 [7–16]	**0.006**	8 [5–12]	8 [7–15]	0.437

*SAH* systemic arterial hypertension, *DM* Diabetes mellitus, *PSQI* Pittsburgh Sleep Quality Index, *WASO *Time awake after sleep onset. Values shown in bold indicate statistical significance (*p* < 0.05)

Table 3 presents clinical outcomes compared over time across the sleep phenotypes, considering the subjective sleep assessment. There was worsening of lung function in both groups, but more pronounced in the ‘short sleep’ phenotype. There was also reduction in light PA, increase in sedentary time and reduction in peripheral muscle strength in the ‘short sleep’ phenotype.

**Table 3:** Comparison of clinical outcomes according to sleep phenotypes based on subjective assessment

Variable	High propensity to sleep + average sleep (*n* = 13)			Short sleep (*n* = 16)		
	A1	A2	p A1 vs. A2	A1	A2	p A1 vs. A2
Sex, Female, *n* (%)	8 (62)	-	**-**	10 (63)	-	**-**
Age, years	70 [67–76]	-	-	67 [61–72]	-	-
Body mass index, kg/m^2^	27 [24–30]	27 [23–31]	0.929	27 [25–31]	28 [24–29]	0.470
Lung function						
FVC, liters	2.41 [2.14–2.86]	2.21 [1.98–2.60]	**0.023**	2.70 [2.31–3.33]	2.38 [2.01–3.01]	**0.004**
FVC, % predicted	84 [67–89]	73 [62–94]	**0.046**	84 [78–98]	75 [59–93]	**0.009**
FEV_1_, liters	1.25 [1.12–1.51]	1.18 [0.95–1.50]	0.195	1.52 [1.24–1.83]	1.52 [0.98–1.74]	**0.030**
FEV_1_, % predict	56 [48–67]	52 [37–70]	0.346	59 [47–78]	56 [40–69]	**0.034**
FEV_1_/ FVC, %	56 [48–62]	58 [44–63]	0.413	56 [45–64]	58 [51–65]	0.360
Body composition						
Fat free mass, kg	34 [27–49]	34 [26–49]	0.424	34 [31–49]	37 [32–58]	0.158
Fat free mass, %	49 [45–70]	48 [47–72]	0.213	55 [48–65]	58 [48–70]	0.060
Fat-free mass index	15 [12–19]	14 [12–19]	0.248	13 [12–19]	15 [13–20]	0.239
Fat mass, kg	32 [20–35]	30 [19–34]	0.091	29 [21–38]	32 [20–37]	0.117
Fat mass, %	51 [30–55]	52 [28–53]	0.213	45 [35–52]	42 [30–52]	0.060
Fat mass index	32 [20–35]	30 [19–34]	0.091	29 [21–38]	32 [20–37]	0.117
Questionnaires						
HADS – A, points	4 [2–9]	5 [4–13]	0.219	12 [4–14]	11 [1–15]	0.166
HADS – D, points	1 [1–7]	2 [1–6]	0.836	9 [5–17]	8 [4–17]	0.346
mMRC, points	1 [0–2]	1 [1–3]	0.058	1 [1–2]	2 [1–3]	0.107
Daily physical activity						
Days of monitoring, days	7 [7–8]	6 [6–7]	0.068	7 [5–7]	6 [4–7]	0.367
Time of use per day, min	840 [794–894]	802 [708–841]	0.173	775 [713–856]	807 [669–1059]	0.408
Sedentary time, min/day	485 [433–542]	452 [378–530]	0.382	421 [283–533]	494 [403–665]	**0.041**
Sedentary time, % of the day	58 [51–66]	60 [49–72]	0.075	55 [34–67]	61 [55–72]	**0.011**
Light PA, min/day	346 [282–410]	288 [200–410]	**0.028**	354 [261–417]	305 [204–422]	0.173
Light PA, % of the day	41 [33–48]	38 [28–48]	0.101	44 [32–62]	38 [26–44]	**0.009**
MVPA, min/day	9 [3–13]	6 [4–14]	0.972	10 [4–19]	9 [6–13]	0.379
MVPA, % of the day	2 [1–3]	3 [2–5]	0.075	3 [1–5]	2 [1–4]	0.100
Functional capacity tests						
4MGS, sec	4 [4–5]	4 [4–5]	0.529	4 [3–5]	4 [4–4]	0.955
TUG, sec	11 [10–12]	12 [10–12]	0.422	11 [9–14]	11 [10–13]	0.363
1-minute STS, repetitions	17 [14–19]	18 [14–20]	0.654	17 [10–22]	15 [13–19]	0.138
Muscle strength						
Handgrip, kgf	25 [20–28]	22 [20–30]	0.349	28 [17–38]	26 [14–36]	0.123
Quadriceps femoris, kgf	33 [26–40]	21 [18–38]	**0.001**	36 [29–48]	25 [18–39]	**0.002**

*FVC* forced vital capacity, *FEV1* forced expiratory volume in first second, *HADS* Hospital Anxiety and Depression Scale, *mMRC* Modified Medical Research Council, *PA* physical activity, *MVPA* Moderate to vigorous physical activity, *4MG*S 4-meter gait speed, *TUG* Timed up and go test, *STS* Sit-to-Stand. Values shown in bold indicate statistical significance (*p* < 0.05)

Table 4 presents clinical outcomes compared over time across sleep phenotypes, considering the objective sleep assessment. In this analysis, 25 individuals were studied due to technical failure of the Actiwatch in four individuals. Results reinforced and expanded on those from the subjective sleep assessment, showing that the ‘short sleep’ phenotype was associated with a marked worsening of lung function, dyspnea, sedentary time, light PA and quadriceps femoris strength.

**Table 4:** Comparison of clinical outcomes according to sleep phenotypes based on objective assessment

Variable	High propensity to sleep + average sleep (*n* = 12)			Short sleep (*n* = 13)		
	A1	A2	p A1 vs. A2	A1	A2	p A1 vs. A2
Sex, female, n (%)	8 (67)	-	**-**	8 (62)	-	**-**
Age, years	70 [61–73]	-	-	67 [61–72]	-	-
Body mass index, kg/m^2^	27 [23–33]	28 [23–33]	0.260	27 [25–29]	28 [24–28]	0.239
Lung function						
FVC, liters	2.43 [2.14–3.14]	2.47 [1.95–2.98]	0.155	2.66 [2.35–3.02]	2.19 [2.04–2.56]	**0.003**
FVC, % predicted	84 [65–93]	73 [62–93]	0.223	85 [84–97]	77 [64–96]	**0.002**
FEV_1_, liters	1.24 [1.02–1.71]	1.29 [0.91–1.71]	0.637	1.47 [1.27–1.68]	1.24 [1.05–1.70]	**0.038**
FEV_1_, % predict	53 [43–67]	51 [36–69]	0.592	64 [49–77]	58 [45–73]	**0.025**
FEV_1_/ FVC, %	51 [44–58]	57 [43–58]	0.083	61 [46–64]	59 [52–64]	0.443
Body composition						
Fat free mass, kg	34 [33–53]	37 [32–58]	0.086	33 [28–43]	35 [30–48]	0.721
Fat free mass, %	56 [49–66]	58 [47–65]	0.139	52 [44–70]	51 [47–70]	0.285
Fat-free mass index	14 [12–20]	14 [13–21]	0.110	14 [12–17]	15 [13–17]	0.799
Fat mass, kg	31 [21–36]	31 [18–42]	0.374	28 [21–36]	30 [20–35]	0.169
Fat mass, %	44 [34–51]	42 [35–53]	0.139	48 [30–56]	49 [30–53]	0.285
Fat mass index	31 [21–36]	31 [18–42]	0.374	28 [21–36]	30 [20–35]	0.169
Questionnaires						
HADS – A, points	5 [2–9]	4 [1–12]	0.607	12 [3–14]	12 [3–16]	0.403
HADS – D, points	4 [1–8]	4 [1–7]	0.527	9 [3–16]	8 [2–17]	0.679
mMRC, points	1 [0–1]	1 [0–3]	0.053	2 [1–2]	2 [2–3]	**0.020**
PADL						
Days of monitoring, days	7 [6–7]	6 [5–7]	0.150	7 [5–8]	6 [5–7]	0.425
Time of use per day, min	815 [756–882]	774 [646–843]	0.347	849 [720–899]	841 [671–1122]	0.600
Sedentary time, min/day	456 [372–567]	449 [370–579]	0.999	485 [317–524]	468 [411–694]	0.152
Sedentary time, % of the day	58 [47–66]	58 [54–71]	0.060	57 [37–66]	60 [53–70]	**0.028**
Light PA, min/day	337 [275–413]	301 [201–378]	**0.041**	349 [289–433]	326 [204–447]	0.221
Light PA, % of the day	41 [33–51]	41 [27–44]	0.060	42 [33–60]	38 [29–46]	**0.033**
MVPA, min/day	8 [4–17]	9 [5–14]	0.999	9 [5–16]	7 [5–14]	0.576
MVPA, % of the day	3 [2–4]	2 [2–4]	0.480	3 [2–5]	4 [2–5]	0.463
Functional capacity tests						
4MGS, sec	4 [3–4]	4 [4–4]	0.374	5 [4–6]	4 [4–5]	0.382
TUG, sec	10 [9–11]	11 [10–12]	0.182	12 [11–15]	11 [10–14]	0.722
1-minute STS, repetitions	17 [14–23]	16 [14–18]	0.582	18 [9–22]	18 [13–21]	0.682
Muscle strength						
Handgrip, kgf	27 [23–34]	26 [22–34]	0.153	25 [15–37]	20 [11–37]	0.306
Quadriceps femoris, kgf	38 [33–48]	31 [21–41]	**0.003**	30 [25–42]	19 [16–32]	**0.003**

*FVC* forced vital capacity, *FEV1* forced expiratory volume in first second, *HADS* Hospital Anxiety and Depression Scale, *mMRC* Modified Medical Research Council, *PA* physical activity, *MVPA* Moderate to vigorous physical activity, *4MGS* 4-meter gait speed, *TUG* Timed up and go test, *STS* Sit-to-Stand. Values shown in bold indicate statistical significance (*p* < 0.05)

An intergroup comparison based on delta values was performed and is presented in the Supplementary Material. Figure 1-S shows that the subjective ‘short sleep’ phenotype presented worse time-related outcomes for sedentary time, both in minutes and % of the day, compared to the “high propensity to sleep + average sleep” phenotype. Some sex-related differences were also identified, with worse outcomes observed in women. Also included in the Supplementary Material are Table 1-S and 2-S, which present the 95% confidence intervals for outcomes related to subjective and objective sleep phenotypes. It is important to note that most of these intervals included zero, indicating the absence of statistically significant differences between the groups.

Further, the mixed repeated-measures ANOVA revealed a significant main effect of time for both light-intensity PA (F(1,27) = 6.272; *p* = 0.019; ηp² = 0.189) and sedentary time (F(1,27) = 11.875; *p* = 0.002; ηp² = 0.305), indicating large effect sizes in subjective sleep phenotypes. There was no significant interaction between time and subjective sleep phenotypes for light-intensity PA (F(1,27) = 0.043; *p* = 0.838; ηp² = 0.002) or sedentary time, although a borderline result was observed for sedentary time (F(1,27) = 3.893; *p* = 0.059; ηp² = 0.126). Additionally, no significant main effect of subjective sleep phenotypes was observed for light-intensity PA (F(1,27) = 0.174; *p* = 0.680; ηp² = 0.006) or sedentary time (F(1,27) = 0.507; *p* = 0.483; ηp² = 0.018).

Because of the absence of comparable studies for calculating sample size previously, the power calculation was conducted using a conservative effect size of 0.5, an alpha of 0.05 and a sample of 29 individuals; thus, the power of the study was 0.74.

## Discussion

To the best of the authors’ knowledge, this is the first study to examine the clinical impact of sleep phenotypes in individuals with COPD over a 1-year follow-up involving both subjective and objective sleep assessments. Results showed that those with a ‘short sleep’ phenotype (based on subjective and objective records) experienced more pronounced worsening of lung function, sedentary behavior, daily PA and peripheral muscle strength than those with the ‘high propensity to sleep + average sleep’ phenotype. Furthermore, the interaction between sleep time and phenotypes has the potential to influence physical inactivity and sedentary behavior. It is important to underline that, due to the limited sample size, these analyses were exploratory and hypothesis-generating, and no correction for multiplicity has been applied. However, we believe that the confidence intervals further reinforce and strengthen the findings.

A decline in lung function was observed in both subjectively derived sleep phenotypes, but with different patterns: the ‘high propensity to sleep + average sleep’ phenotype showed worsening only in FVC, while the ‘short sleep’ phenotype declined in both FVC and FEV₁. In objectively derived phenotypes, this difference was more evident, with lung function decline occurring exclusively in the ‘short sleep’ phenotype. Since FEV₁ reflects disease severity and predicts exacerbations and mortality [15], these findings highlight the clinical relevance of inadequate sleep. Although previous studies have linked poor sleep to worse lung function [16], airflow limitation, and gas exchange impairment, the underlying mechanisms remain nuclear [17].

The decline in light-intensity PA and the increase in sedentary behavior observed in this study are concerning for individuals with COPD, as moderate-intensity PA targets are often difficult to achieve [18], making light PA a more feasible alternative [19]. This reduction in light activity has been previously reported and may occur even without intervention [20]. The ‘short sleep’ phenotype may contribute to this decline through physiological and behavioral mechanisms, such as impaired recovery, increased fatigue, autonomic dysfunction, inflammation, and greater perception of dyspnea and effort. These factors may reduce exercise tolerance and promote sedentary behavior, suggesting a bidirectional relationship in which insufficient sleep and physical inactivity contribute to functional decline over time [21].

Many efforts are currently focused on investigating sedentary behavior in individuals with COPD. Increased sedentary behavior is already recognized as being closely linked to poorer clinical outcomes and reduced life expectancy. Additionally, sedentary behavior in individuals with COPD is known to be influenced by various factors, including sleep [22]. The present results add even more nuances to this scenario because of the different sedentary behaviors between the phenotypes in the inter and intragroup analyses, in addition to the interaction in time and sleep phenotypes. The theories regarding this association are varied and may interact synergistically. Poor sleep is correlated with heightened awareness of nocturnal symptoms and increased daytime sleepiness, which can diminish an individual’s motivation, leading to reduced engagement in activities and increased sedentary behavior. Another hypothesis suggests that poor sleep quality may adversely impact energy metabolism, diminishing the energy available for PA and promoting sedentary behavior [23].

A significant decline in quadriceps strength was observed in both sleep phenotypes, consistent with previous evidence linking sleep and peripheral muscle strength in COPD [24]. Both short and long sleep durations, as well as poor sleep quality, have been associated with impaired muscle function [25]. Even in the ‘high propensity to sleep + average sleep’ phenotype, many individuals did not fully meet sleep recommendations, which may have influenced the results. This relationship may be explained by the effects of sleep on muscle protein synthesis and degradation, mediated by hormonal and metabolic factors, in addition to related influences such as physical inactivity [5] and psychological aspects.

Although the minimal clinically important difference (MCID) for light-intensity PA and sedentary time in individuals with COPD has not yet been established, small reductions in light PA or increases in sedentary time may still have functional impact. For muscle strength, a decrease of approximately 10–15% in maximal voluntary isometric contraction of the quadriceps is considered clinically significant, indicating marked functional loss even over relatively short periods [26].

Although this study did not incorporate a standardized assessment of sleep-disordered breathing, we cannot overlook obstructive sleep apnea (OSA). This condition is the most prevalent respiratory disorder in COPD [27, 28] and is linked to elevated WASO values, numerous nocturnal awakenings and snoring [29]. We believe that individuals with COPD and concurrent OSA would fall within the ‘high propensity to sleep + average sleep’ phenotype, due to their characteristics and the need to remain in bed for extended periods to “suppress” excessive awakenings, which reflect the recurrent collapse of the upper airway during sleep. The ‘short sleep’ phenotype may be linked to nocturnal intermittent hypoxemia, resulting in sympathetic overload that operates through hyperexcitation of chemoreceptors and disrupts sleep-related processes [30]. Finally, the ‘average sleep’ phenotype may be linked to protective factors for OSA, including lung hyperinflation and low BMI [31]. It is important to emphasize that for the moment these are hypotheses, which may or may not be confirmed by future studies investigating the impact of polysomnography-based sleep phenotypes in individuals with COPD.

This study may add to the knowledge gained from the previous investigation by Razjouyan et al. [10] involving sleep phenotypes in individuals with COPD. While Razjouyan et al. [10] focused on identifying sleep phenotypes associated with mortality in individuals with COPD, we emphasized the relationship between sleep phenotypes and clinical outcomes relevant to the disease. Both studies open room for further research to address important gaps in this field.

This study has both strengths and limitations. Measurement of sleep by two different methods offers a comprehensive assessment of sleep that is not common in the literature. Furthermore, the diversity of outcomes analyzed in a standardized and objective manner, along with the 12-month follow-up enhances the study’s novelty. However, the small sample size is a limitation that, while sufficient to indicate various significant differences, may suffer from a lack of study power in given variables and may affect the external validity of the study. The combination of ‘high propensity to sleep’ and ‘average sleep’ phenotypes into a single category, owing to the small subgroups, should also be acknowledged as a limitation. The lack of measurement of sleep-related breathing disorders, especially OSA, is an important factor that may also have influenced the present results. Furthermore, the present sample primarily included individuals with moderate-to-severe airflow obstruction, which may prevent the generalization of the results to those with mild or very severe obstruction. We suggest that future research focus on investigating various sleep phenotypes in larger and more heterogeneous populations, with longer follow-up periods.

In conclusion, individuals with COPD and a ‘short sleep’ phenotype experience significant declines in lung function, daily PA, sedentary behavior, dyspnea and peripheral muscle strength over a 12-month follow-up. Furthermore, significant interactions were observed between sleep phenotypes and both light-intensity physical activity and sedentary behavior. Identifying strategies to enhance sleep quality, as a modifiable factor, may be essential in the management and comprehensive care of this disease. Despite this, we recognize the importance of investigating the three sleep phenotypes separately in future studies, as well as examining potential changes in sleep phenotypes over time and their clinical impact on individuals with COPD.

## Supplementary material

Below is the link to the electronic supplementary material.

Supplementary File 1 (DOCX 114 KB)

## Data Availability

The data that support the findings of this study are not openly available due to reasons of sensitivity and are available from the corresponding author upon reasonable request.

## Declarations

### Presentation at a conference

Not presented.
