# Supplementary material for: Sleep phenotypes and clinical outcomes in individuals with chronic obstructive pulmonary disease: A cohort study
Source: Sleep Breath. 2026 Apr 25;30(2):140. doi: 10.1007/s11325-026-03683-2 (PMC13110220; doi:10.1007/s11325-026-03683-2)
Supplement: Supplementary file 1 — (DOCX 114 KB) [file 11325_2026_3683_MOESM1_ESM.docx]

**TITLE:** Sleep phenotypes and clinical outcomes in individuals with chronic obstructive pulmonary disease: a cohort study.

**Supplementary material**

*Clinical data*

A structured questionnaire was employed at A1 for the initial gathering of personal, clinical and demographic information. The questions addressed smoking history, associated comorbidities, past medications, previous exacerbations and the need for oxygen therapy, along with the systematic collection of all personal contacts and those of two family members for follow-up.

At A2, questions were asked about changes in personal and demographic data, changes in smoking habits, current medications, exacerbations in recent months, onset of new comorbidities and individual perceptions of changes in sleep patterns, physical activity, physiotherapy or participation in other research.

*Lung function*

Lung function assessment was performed using spirometry and measurement of lung volumes using a body plethysmograph (Vmax Carefusion, Germany). The techniques were performed according to international guidelines^(1)^, and reference values used were those for the Brazilian population^(2)^.

*Body composition*

Body composition was assessed by bioelectrical impedance analysis (Biodynamics 310TM; Biodynamics Corp, USA). Participants were instructed to avoid exercising for at least 12 hours before the test and refrain from the ingestion of coffee, tea, chocolate, or alcoholic beverages. Body composition assessment was performed in a single measurement with patients lying in the supine position during the morning. Participants fasted for at least 4 hours before the test and their bladders were emptied immediately before the evaluation.

Fat-free mass was calculated using a specific formula derived for patients with COPD^(3)^. Fat mass was calculated by subtracting fat-free mass from body weight. Considering differences in body surface, fat-free mass and fat mass were divided by height squared to compose the fat-free mass index and fat mass index, respectively.

*Daily physical activity (PA)*

The assessment of daily PA was conducted using the validated ActiGraph wGT3X-BT physical activity monitor (Pensacola,USA)^(4)^. Participants wore the small device on an elastic belt around the waist, positioned towards the right knee, during waking hours for 7 consecutive days (including weekends), removing it only for water-related activities. Activity intensity was categorized using the following cut-off points: sedentary behavior (≤1.5 METs), light-intensity physical activity (LIPA; 1.5–2.9 METs), and moderate-to-vigorous physical activity (MVPA; ≥3 METs). The classification was performed using the algorithm developed by Freedson VM3. During the days the devices were worn, participants were instructed to maintain a diary detailing the moments they removed the device, as well as their bedtime, wake-up time, bath time and any additional relevant information. An assessment was deemed valid when there were at least 4 valid days, and a valid day was defined as at least 480 minutes of monitor use per day^(5)^. In this study, all patients had measurements taken on weekdays and weekends, and there were no exclusions due to incorrect use of the monitor.

*Handgrip strength*

Handgrip strength of both hands was assessed with the use of a hydraulic hand dynamometer (SH50011; Saehan Corporation,Changwon,South Korea). Individuals were in a seated position with their arms unsupported, shoulders in a neutral position along the body, elbows flexed to 90° and wrists in neutral position. Six seconds of contraction and 30 seconds of rest between attempts were adopted; five attempts were made for each hand, unless the last value was the highest, in which case more attempts were performed until the last was no longer the highest. The highest value of the self-reported dominant hand was used in the analysis.

*Quadriceps femoris strength*

The peak muscle strength of the knee extensors (i.e., quadriceps femoris) was assessed using a 200 kgf traction-compression dynamometer (EMG System, Brazil), with signal captured by the signal acquisition system (EMG System, Brazil). The dynamometer was secured to a multi-station weight training machine (CRW 1000; Brazil), and the evaluation angle was set at 30º of knee extension.

Participants were seated upright with back support, maintaining 90º of hip flexion and resting their hands on the respective lower limbs. This evaluation involved the maximum isometric contraction of the quadriceps, which lasted six seconds. A minimum of four measurements and a maximum of fifteen measurements were taken for each evaluated lower limb, and strength of the self-reported dominant leg was used in the analysis, provided that the two highest values differed by less than 5%^(6)^. The test was conducted by a single, properly trained evaluator who provided standardized verbal encouragement throughout the maneuver.

*Functional capacity tests*

Three tests were used to assess functional exercise capacity. For the 4-meter gait speed (4MGS), subjects were instructed to walk at their usual speed along a 4-meter course. The time taken for the test was recorded in seconds using a stopwatch. Two attempts were conducted by the same evaluator, and the best attempt (i.e., the shortest duration) was used for analysis. Participants were allowed to rest between attempts if needed^(7).^

In the timed up-and-go test (TUG), individuals were instructed to stand up from their chair, walk a distance of 3 meters at a comfortable and safe pace, turn around and walk back to the chair to sit down again. The time taken to complete the test (in seconds) was recorded using a stopwatch and served as the primary outcome for analysis. Two attempts were conducted by the same evaluator, and the best attempt (i.e., the shortest duration) was used for analysis. Participants were allowed to rest between attempts if necessary^(8)^.

Finally, in the 1-minute Sit-to-Stand (STS) test, individuals were instructed to perform sitting and standing movements during one minute without using their upper limbs for assistance. The stopwatch was started at the evaluator's voice command, who counted the number of times the individual stood up completely from the chair. Immediately before and after the STS, values of peripheral oxygen saturation, heart rate, systolic and diastolic blood pressure were recorded, as well as symptoms of dyspnea and fatigue in the lower limbs using the modified Borg scale. Two attempts were conducted by the same evaluator, and the best attempt (i.e., the highest number of repetitions) was selected for analysis. Participants were required to rest between attempts until their vital signs returned to baseline values^(9)^.

*Anxiety and depression*

The Hospital Anxiety and Depression Scale (HADS)^(10)^, in its validated Portuguese version^(11)^, was used to evaluate anxiety and depression. This scale comprises 14 items, with 7 focused on anxiety and 7 focused on depression. Each item is scored from 0 to 3, allowing for a maximum score of 21 points per subscale. The questionnaire was employed solely for quantification purposes, without any diagnostic application of the instrument.

*Dyspnea*

The Modified Medical Research Council (mMRC) scale was used to assess dyspnea. The scale comprises 5 options in which the individual chooses to what extent the dyspnea impairs his/her daily functioning^(12)^.

**RESULTS**

Regarding intergroup comparisons, Figure 1-S shows that the ‘short subjective sleep’ phenotype had significantly worse results than the ‘high propensity to sleep + average sleep’ phenotype in terms of sedentary time, both in minutes (p=0.042) and as a percentage of the day (p=0.046). No further significant differences were found regarding the deltas of the other study outcomes. When analyzed by sex, in women the ‘short sleep’ phenotype was also worse than the group with ‘high propensity to sleep + average sleep’ phenotype concerning the deltas of sedentary time, both in minutes (300[23 to 371] *versus* -6[-78 to 48], respectively; p=0.031) and as a percentage of the day (12[6 to 25] *versus* 4[0 to 11], respectively; p=0.042). In men, this significant difference in deltas occurred in the STS, once again with the ‘short sleep’ phenotype showing worse results than the ‘high propensity to sleep + average sleep’ phenotype ( -2 [-5 to 1] *versus* 2[-1 to 4], respectively; p=0.046).

Concerning the deltas (i.e., A2 minus A1) compared across phenotypes based on the objective sleep assessment, no significant differences were found. Consonantly, there were no significant differences also when analyzed by sex.

Figure 1-S.


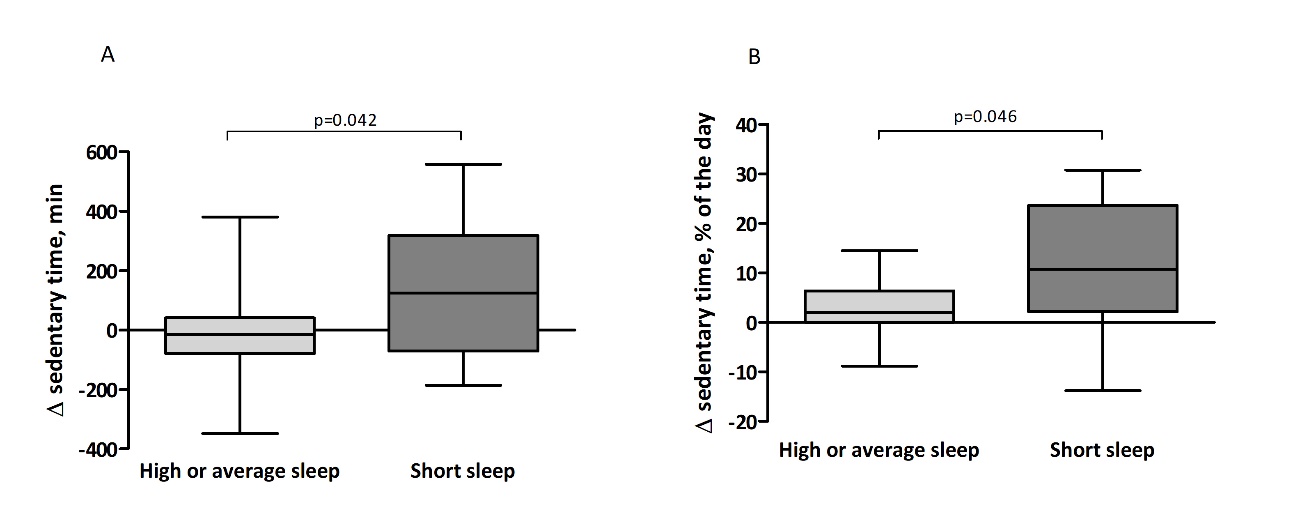


Figure 1 - S: Comparison of sedentary time deltas between subjective sleep phenotypes, where delta corresponds to A2 minus A1 (or assessment after 12 months minus baseline). Positive deltas indicate worsening (i.e., increase) sedentary time, whereas deltas close to zero indicate no marked change. "A" depicts the delta of sedentary time in minutes (median [25-75% IQR]: -14 [-78 to 41] for the ‘high propensity to sleep + average sleep’ phenotype, and 109 [-52 to 318] for the ‘short sleep’ phenotype); "B" depicts the delta of sedentary time in % per day (2 [0 to 6] for the ‘high propensity to sleep + average sleep’ phenotype, and 10 [1 to 23] for the ‘short sleep’ phenotype).

Finally, as presented in Tables 1-S and 2-S, the 95% confidence intervals for the differences in clinical, functional, body composition, physical activity, and muscle strength outcomes between subjective and objective sleep phenotypes included zero for most variables. This indicates the absence of statistically significant differences between phenotypes over the follow-up period. Additionally, the relatively wide range of some intervals suggests individual variability in responses, but without consistent evidence of worsening or improvement associated with a specific phenotype. These results reinforce that, overall, sleep phenotypes, particularly when assessed objectively, were not associated with significant changes in the clinical outcomes evaluated in this cohort.

**Table 1-S: Confidence interval for outcomes related to subjective sleep phenotypes.**

| **Variable** | **95% CI – lower** | **95% CI – upper** |
| --- | --- | --- |
| Age, years | - 3.000 | 10.000 |
| Body mass index, kg/m^2^ | - 4.190 | 3.990 |
| **Lung function** |  |  |
| FVC, liters | - 0.710 | 0.220 |
| FVC, % predicted | - 15.000 | 14.000 |
| FEV_1_, liters | - 0.570 | 0.170 |
| FEV_1_, % predict | - 17.000 | 15.000 |
| FEV_1_/ FVC, % | - 12.000 | 4.000 |
| **Body composition** |  |  |
| Fat free mass, kg | - 14.039 | 6.384 |
| Fat free mass, % | - 11.892 | 11.095 |
| Fat-free mass index | - 4.060 | 2.836 |
| Fat mass, kg | - 10.683 | 6.711 |
| Fat mass, % | - 11.095 | 11.892 |
| Fat mass index | - 10.683 | 6.711 |
| **Questionnaires** |  |  |
| HADS – A, points | - 7.000 | 3.000 |
| HADS – D, points | - 13.000 | - 1.000 |
| mMRC, points | - 2.000 | 0.000 |
| **Daily physical activity** |  |  |
| Days of monitoring, days | 0.000 | 2.000 |
| Time of use per day, min | - 214.464 | 112.900 |
| Sedentary time, min/day | - 179.286 | 68.306 |
| Sedentary time, % of the day | - 9.934 | 8.234 |
| Light PA, min/day | - 112.869 | 82.548 |
| Light PA, % of the day | - 8.003 | 10.060 |
| MVPA, min/day | - 5.800 | 2.286 |
| MVPA, % of the day | - 0.344 | 2.384 |
| **Functional capacity tests** |  |  |
| 4MGS, sec | - 0.720 | 0.520 |
| TUG, sec | - 1.560 | 1.320 |
| 1-minute STS, repetitions | - 2.000 | 7.000 |
| **Muscle strength** |  |  |
| Handgrip, kgf | - 14.000 | 8.000 |
| Quadriceps femoris, kgf | - 8.460 | 8.680 |

FVC: forced vital capacity; FEV1: forced expiratory volume in first second; HADS: Hospital Anxiety and Depression Scale; mMRC: Modified Medical Research Council ; PA: physical activity; MVPA: Moderate to vigorous physical activity ; 4MGS: 4-meter gait speed; TUG: Timed up and go test; STS: Sit-to-Stand.

**Table 2-S: Confidence interval for outcomes related to objective sleep phenotypes.**

| **Variable** | **95% CI – lower** | **95% CI – upper** |
| --- | --- | --- |
| Age, years | - 4.000 | 9.000 |
| Body mass index, kg/m^2^ | - 4.180 | 6.380 |
| **Lung function** |  |  |
| FVC, liters | - 0.340 | 0.660 |
| FVC, % predicted | - 17.000 | 15.000 |
| FEV_1_, liters | - 0.420 | 0.360 |
| FEV_1_, % predict | - 24.000 | 12.000 |
| FEV_1_/ FVC, % | - 13.000 | 5.000 |
| **Body composition** |  |  |
| Fat free mass, kg | - 7.209 | 14.143 |
| Fat free mass, % | - 10.305 | 12.494 |
| Fat-free mass index | - 3.335 | 4.659 |
| Fat mass, kg | - 6.949 | 11.187 |
| Fat mass, % | - 12.494 | 10.305 |
| Fat mass index | - 6.949 | 11.187 |
| **Questionnaires** |  |  |
| HADS – A, points | - 11.000 | 0.000 |
| HADS – D, points | - 12.000 | 1.000 |
| mMRC, points | - 2.000 | 0.000 |
| **Daily physical activity** |  |  |
| Days of monitoring, days | - 1.000 | 1.000 |
| Time of use per day, min | - 275.268 | 51.100 |
| Sedentary time, min/day | - 218.238 | 50.871 |
| Sedentary time, % of the day | - 10.232 | 9.917 |
| Light PA, min/day | - 150.810 | 82.548 |
| Light PA, % of the day | - 8.153 | 10.595 |
| MVPA, min/day | - 4.542 | 5.825 |
| MVPA, % of the day | - 2.613 | 0.408 |
| **Functional capacity tests** |  |  |
| 4MGS, sec | - 1.010 | 0.180 |
| TUG, sec | - 3.060 | 1.210 |
| 1-minute STS, repetitions | - 6.000 | 4.000 |
| **Muscle strength** |  |  |
| Handgrip, kgf | - 7.000 | 17.000 |
| Quadriceps femoris, kgf | 0.440 | 18.700 |

FVC: forced vital capacity; FEV1: forced expiratory volume in first second; HADS: Hospital Anxiety and Depression Scale; mMRC: Modified Medical Research Council ; PA: physical activity; MVPA: Moderate to vigorous physical activity ; 4MGS: 4-meter gait speed; TUG: Timed up and go test; STS: Sit-to-Stand.

**REFERENCES**

1. Miller MR, Crapo R, Hankinson J, Brusasco V, Burgos F, Casaburi R, et al. General considerations for lung function testing. Eur Respir J. 2005;26(1):153-61.

2. Pereira CA, Sato T, Rodrigues SC. New reference values for forced spirometry in white adults in Brazil. J Bras Pneumol. 2007;33(4):397-406.

3. Steiner MC, Barton RL, Singh SJ, Morgan MD. Bedside methods versus dual energy X-ray absorptiometry for body composition measurement in COPD. Eur Respir J. 2002;19(4):626-31.

4. Rabinovich RA, Louvaris Z, Raste Y, Langer D, Van Remoortel H, Giavedoni S, et al. Validity of physical activity monitors during daily life in patients with COPD. Eur Respir J. 2013;42(5):1205-15.

5. Demeyer H, Burtin C, Van Remoortel H, Hornikx M, Langer D, Decramer M, et al. Standardizing the analysis of physical activity in patients with COPD following a pulmonary rehabilitation program. Chest. 2014;146(2):318-27.

6. Hopkinson NS, Tennant RC, Dayer MJ, Swallow EB, Hansel TT, Moxham J, et al. A prospective study of decline in fat free mass and skeletal muscle strength in chronic obstructive pulmonary disease. Respir Res. 2007;8(1):25.

7. Kon SS, Canavan JL, Nolan CM, Clark AL, Jones SE, Cullinan P, et al. The 4-metre gait speed in COPD: responsiveness and minimal clinically important difference. Eur Respir J. 2014;43(5):1298-305.

8. Marques A, Cruz J, Quina S, Regêncio M, Jácome C. Reliability, Agreement and Minimal Detectable Change of the Timed Up & Go and the 10-Meter Walk Tests in Older Patients with COPD. Copd. 2016;13(3):279-87.

9. Morita AA, Bisca GW, Machado FVC, Hernandes NA, Pitta F, Probst VS. Best Protocol for the Sit-to-Stand Test in Subjects With COPD. Respir Care. 2018;63(8):1040-9.

10. Zigmond AS, Snaith RP. The hospital anxiety and depression scale. Acta Psychiatr Scand. 1983;67(6):361-70.

11. Botega NJ, Bio MR, Zomignani MA, Garcia C, Jr., Pereira WA. [Mood disorders among inpatients in ambulatory and validation of the anxiety and depression scale HAD]. Rev Saude Publica. 1995;29(5):355-63.

12. Sunjaya A, Poulos L, Reddel H, Jenkins C. Qualitative validation of the modified Medical Research Council (mMRC) dyspnoea scale as a patient-reported measure of breathlessness severity. Respir Med. 2022;203:106984.
